# Current Loss-of-Function Mutations in the Thyrotropin Receptor Gene: When to Investigate, Clinical Effects, and Treatment

**DOI:** 10.4274/Jcrpe.864

**Published:** 2013-03-01

**Authors:** Alessandra Cassio, Annalisa Nicoletti, Angela Rizzello, Emanuela Zazzetta, Milva Bal, Lilia Baldazzi

**Affiliations:** 1 Department of Gynaecologic, Obstetric and Paediatric Sciences, S.Orsola-Malpighi Hospital, University of Bologna, Bologna, Italy

**Keywords:** Thyrotropin receptor gene mutations, congenital hypothyroidism, Neonatal screening, Subclinical hypothyroidism

## Abstract

Thyroid-stimulating hormone receptor (TSHR) loss-of-function (LOF) mutations lead to a wide spectrum of phenotypes, ranging from severe congenital hypothyroidism (CH) to mild euthyroid hyperthyrotropinemia. The degree of TSH resistance depends on the severity of the impairment of the receptor function caused by the mutation and on the number of mutated alleles In this review data about genotype-phenotype correlation and criteria for clinical work-up will be presented and discussed. Complete TSH resistance due to biallelic LOF TSHR mutations must be suspected in all patients with severe not syndromic CH and severe thyroid hypoplasia diagnosed at birth by neonatal screening. Partial forms of TSH resistance show a more heterogeneous hormonal and clinical pattern . In these cases TSH serum levels are above the upper limit of normal range for the age but with a very variable pattern, free thyroxine (T4) concentrations are within the normal range and thyroid size can be normal or hypoplastic at ultrasound scan. An early substitutive treatment with L-T4 must be mandatory in all patients with severe CH due to complete uncompensated TSH resistance diagnosed at birth by neonatal screening. The usefulness of substitutive treatment appears much more controversial in patients with subclinical hypothyroidism due to partial TSH resistance in whom the increased TSH concentration should be able to compensate the mild functional impairment of the mutant receptor. Together with standard criteria we recommend also an accurate clinical work-up to select patients who are candidates for a LOF TSHR mutation.

**Conflict of interest:**None declared.

## INTRODUCTION

Congenital hypothyroidism (CH) is the most common endocrine disorder in infancy. It shows a considerable variability in etiopathogenesis and clinical aspects. Among these various disorders, resistance to thyroid-stimulating hormone (TSH) has been defined as a genetic disease, characterized by TSH serum levels above the normal range, normal or reduced thyroid hormone concentrations, normal sized or hypoplastic thyroid gland, in absence of anti-thyroid antibodies. Recently, the OMIM database for genetic disease (at the website www.ncbi.nlm.nih.gov) classified TSH resistance among the six forms of nongoitrous CH (CHNG) and loss-of-function (LOF) mutations in the TSH receptor (TSHR) gene were identified as the most frequent cause of TSH resistance (Table 1) (1,2).

This review will focus on the TSH resistance due to LOF TSHR gene mutations. Data about the spectrum of the TSHR gene mutations, genotype-phenotype correlation, phenotypic variability, and criteria for clinical work-up will be presented and discussed with the help of recent studies on TSHR gene analysis in large populations.

**TSHR Physiology **

**Figure 1:**
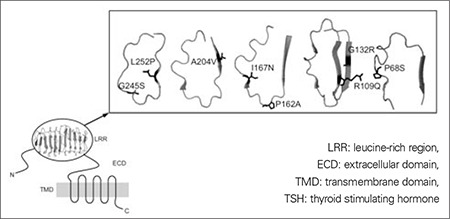
Schematic representation of TSH receptor, LRR domain ispresent in 3D structure (PDB ID 3G04); b)Structure of the mutatedresidues; orientation of functional groups is showed (hydophobic P68,P162, I167, A204, L252; with positive charge R109); (3D structuresperformed with software PyMOL T Molecular Graphics System, Version0.95 by DeLano Scienfic LLC)

TSHR encoded by the TSHR gene (chromosome 14q31) (3) is a G-protein-coupled receptor with a seven-transmembrane domain (TMD) activating the classical G-protein-coupled effectors, adenylatecyclase (AC) and phospholipase C (PLC) and a large extracellular domain (ECD) responsible for high-affinity hormone binding (Figure 1). TSHR ECD is encoded by the first 9 exons and part of exon 10, whereas TMD and intracellular domain are encoded by the exon 10. 

The TSHR ECD contains a leucine-rich region (LRR) involved in hormone binding specificity and two cysteine-rich domains that would contribute to the 3D structure of the ECD and are involved in the maintenance of the two-subunit structure of the receptor. Between the LRR region and the TMD, there is a structural linker (hinge region) probably involved in structural conformation

There are several pieces of evidence indicating that TSHR is present as dimers or higher-order oligomers on the thyroid cell surface. TSHR oligomerization is an early event in receptor maturation and it may play an important role in receptor trafficking and targeting to the cell membrane (2).

**Spectrum of LOF TSHR Gene Mutations**

To date, more than 60 mutations in the TSHR gene have been described in association with different degrees of TSH resistance (Table 2, continued 1, continued 2,) (4,5,6,7,8,9,10,11,12,13,14,15,16,17,18, 19,20,21,22,23,24,25,26,27,28,29,30,31,32,33,34,35,36, 37,38,39,40,41,42). The mutations are all point mutations, such as base substitutions and small deletions/insertions that cause missense (the most frequent), nonsense and frame shift mutations. These mutations are distributed all along the receptor sequence, without any hot spot, the only preserved portion being the C-terminal intracytoplasmatic region. Large deletions involving the entire gene are not reported, but recently, a single exon deletion was reported involving exon 2 (21). 

Recently, Tao (43) suggested a classification of GPRCs gene mutations in five classes according to their functional consequences, namely, class I: defective receptor biosyntesis; class II: defective trafficking to the cell surface; class III: defective ligand binding; class IV: defective receptor activation; class V: mutant with no known defects. In particular, the introduction of this fifth class underlines that some of the structure-function relationships of the GPCRs remain to be assessed.

The mutations in the TSHR gene that cause an absence or strong reduction of the protein product, such as frame-shift, nonsense and splicing mutations, usually affect the amount of the receptor expressed on the plasma membrane with consequent severe impairment of the protein function. Fourteen of 25 patients reported in the literature (Table 2, continued 1, continued 2,) with these three types of mutation showed some degree of thyroid hypoplasia.

The functional effects of the other TSHR gene missense mutations range from a slight alteration in biological function of the receptor to a condition of pseudo-dominance, in which a mutated receptor is able to alter the function of the normal receptor. 

Among the four missense mutations reported in the N-terminal region of ECD (exon 1), two (P27T and E34K) showed a slight activity reduction in functional studies, while the other two showed a severe loss of function (34,35,36,37,38,39,40,41,42,43,44,45,46,47). In particular, C41S mutation is able to exert in vitro a dominant negative effect due to the intracellular entrapment of wild-type receptor by oligomerization with TSHR mutant (44). This molecular mechanism should be able to explain the occurrence of TSH resistance in some cases with heterozygous LOF mutations. 

Eleven mutations map in the LRR domain (Figure 1), whose 3D structure was first available from the crystallized LRR domain of ribonuclease inhibitor protein (47). Recently, also the TSHR LRR crystal structure was determined (48) and this confirmed more accurately the role of the single residues and correlated these results with functional studies. The first correlation between 3D data and functional assay was reported for P162A and I167N mutations (49). In particular, the mild effect of the P162A mutation was confirmed by the fact that spatial orientation of the hydrophobic ring of proline 162 does not contribute to form the hydrophobic core typical of LRR, as made by isoleucine 167. Instead, arginine 109, involved in hydrogen bonds and strong Van der Walls interaction with ligand, is substituted by a polar glutamine (R109Q) that may diminish but not lose the interaction with the ligand, thus corresponding with the loss of almost 50% of binding activity in functional assay (15).

In total, 33 subjects are reported with mutations in the LRR domain, of whom 20 are monoallelic-only four mutations (G132R, I167N, P252L) showed a loss of function both in cAMP production and binding activity (26,28,31), whereas the other mutations showed a strong reduction in binding activity, due to a minor expression of the receptor in cell membrane rather than the amino acidic change, with only a slight reduction in cAMP production. This highlights a possible limit of in vitro methods to evaluate TSHR biological activity.

The last five ECD mutations map in the so-called hinge region that plays a role in structural conformation and undergoes post-transductional modifications. In this group, the mutations R310C (32) and C390W (13) are associated with defective binding and cAMP generation and even increased basal constitutive ligand-independent activity. This finding may confirm the hypothesized inhibitory effect of the ECD on a noisy TMD and it also explains the compensated TSH resistance observed in affected patients (2). The mutation D410N has been reported as associated with normal binding but defective cAMP generation, findings which confirm the role of the hinge region in conveying the signal from the ECD to the TMD (13).

Finally, most of the missense mutations lie in TMD, involved in signal transduction through Gs protein interaction and dimerization. As recently reported, it is assumed that the biochemical function of the TSHR is not guaranteed only by the cAMP generation via Gα proteins (50,51,52). The identification of two mutated TSHRs with normal cAMP generation and impaired IP3 generation via Gq proteins (M527T and L653V) indicated that the biological activity of some TMD mutations can be more correctly measured through IP3 accumulation (8,42). An impairment of the PLC-IP3-Ca2+ pathway affects the iodine uptake, which is not always investigated in clinical studies, resulting in a possible underestimation of this phenotypic sign. Further studies have confirmed this finding (5,10), and some authors have proposed a new definition: “nonclassic” TSH resistance, a dyshormonogenesis- like variant in which the predominant effect of the mutation is the impairment of Gq-mediated signal (10). 

**Genotype-phenotype Correlations**

TSHR LOF mutations lead to a wide spectrum of phenotypes, ranging from severe CH to mild euthyroid hyperthyrotropinemia. The sensitivity of thyroid tissue to TSH stimulation can be fully lost with severe impairment of thyroid function and growth despite an enhanced TSH secretion or partially conserved with compensatory effect of the elevation of TSH levels (complete or partial TSH resistance) (2).

The degree of TSH resistance depends on the severity of the impairment of the receptor function caused by the mutation and on the number of mutated alleles (2). When both alleles carry mutant receptors with complete lack of function, usually, the result is severe CH with a hypoplastic thyroid gland (uncompensated TSH resistance). Less severe LOF mutations, mainly recessively inherited, can manifest as mild/borderline forms of hypothyroidism in which an appropriate increase in TSH serum levels can compensate the reduced sensitivity of the thyroid (partially or fully compensated TSH resistance). Recently, some heterozygous LOF mutations in the TSHR have been described associated with non-autoimmune subclinical hypothyroidism (NASH) and normal sized/hypoplastic gland; in the affected subjects, the defect was dominantly inherited, cosegregating with monoallelic LOF mutations (Table 2, continued 1, continued 2,). 

However, phenotypic variability is a characteristic of TSHR gene mutations. The same mutation can be associated with different thyroid function in different subjects or in the members of the same family, and thyroid hypoplasia, which is preferably associated with null mutations in homozygous state, is found also in patients carrying monoallelic mutations. One emblematic mutation correlating with this phenotypic variability is the P162A mutation, reported in different families (Table 2, continued 1, continued 2,) both in homozygous and in heterozygous state. Not all the patients homozygous for P162A mutation have severe CH, and among the heterozygous subjects, a great variability has been reported in the range of TSH serum levels and in the frequency of thyroid hypoplasia. A very similar situation is found for the R450H mutation, a recurrent mutation in the Japanese population, where a heterozygous subject showed TSH levels even greater that a homozygous subject (35). 

Therefore, these findings suggest that other factors, such as genetic heterogeneity, such as digenic inheritance (23,25), limits in TSHR gene analysis methods (other gene regions generally not included in analysis as promoter, 3’UTR, introns, cis enhancers, or the search for deletions/complex rearrangements involving the entire gene), and environmental factors (different thyroid hormone requirement at different ages, different iodide supply, effect of acquired thyroid disease) can contribute to phenotypic variability. 

Most likely, the pediatric age of patients with NASH examined in our study (8) could explain at least in part the higher prevalence of mutated alleles (11/38; 29%) that we found compared with adult patients examined by Tonacchera et al (2/73; 2.7%) (31). In fact, the increased needs due to growth and development in childhood could promote the expression of NASH phenotype in patients with only minimally impaired thyroid function and negative results at newborn screening. Mizuno et al (53) fhomozound low serum fT4 levels in one patient with homozygous R450H mutation in adolescence, although his thyroid hormones had been within the normal range in early infancy. In many studies, patients affected by either ygous or heterozygous mutations of TSHR gene with fully or partially compensated TSH resistance could show with time an uncompensated resistance in association with development of autoimmune thyroid disease (AITD) (8,24,29).

**Diagnostic and Therapeutic Work-up**

**When to Investigate**

The great phenotypic variability makes often difficult the diagnostic approach to TSH resistance due to LOF TSHR mutations. Table 3 shows the main clinical, hormonal and ultrasound (US) criteria useful to select the patients in whom the diagnosis may be suspected. 

First of all, we have to point out that LOF TSHR mutations should be differentiated from other pathological mechanisms able to cause similar clinical features. The secretion of bioinactive TSH with conserved immuno-reactivity can be hypothesized, but no such cases have been reported (2). Mutations in the gene encoding for G-protein (GNAS1) have been reported as cause of a more generalized form of hormone resistance [defined as pseudo-hypoparathyroidism type 1a (PHP1a)] with expression not only in thyroid but also in target tissues of the other involved hormones [kidney for parathyroid hormone (PTH), gonads for FSH/LH and pituitary for GHRH] (54). Therefore, in these cases, a phenotype with high PTH levels, osteodystrophy and other typical features can be helpful for differential diagnosis. At last, thyroid hypoplasia can be caused by some defects in transcription factors, such as NKX2.1 and PAX8 (55). These factors, differently from TSHR, are expressed in tissues other than thyroid (CNS, lung and kidney) and their mutations can determine the so-called syndromic CH (53).

Complete TSH resistance due to biallelic LOF TSHR mutations must be suspected in all patients with severe non-syndromic CH and severe thyroid hypoplasia diagnosed at birth by neonatal screening. The prevalence of this form of CH varies among different screening programs, but it is considered a rare condition. Likely, its real (effective, actual) incidence can be underestimated due to its being strongly dependant on the experience of the operator for a correct US diagnosis. In fact, a mistaken diagnosis of athyreosis should be made at scintigraphy due to impaired radioiodine uptake by the very small amount of thyroid tissue (39). The detection of a normal thyroglobulin serum level can be useful for differential diagnosis. Genetic analysis of the family in affected subjects may be able to reveal some heterozygous relatives with biochemical features of partial resistance (2).

The diagnostic approach becomes very puzzling in partial forms of TSH resistance due to LOF TSHR mutations, since these cases show a more heterogeneous hormonal and clinical pattern (Table 3). In these cases, TSH serum levels are above the upper limit of normal range for age but show a very variable pattern, free thyroxine (fT4) concentrations are within the normal range and thyroid size can be normal or hypoplastic at US scan. The prevalence data reported by neonatal screening programs appear discordant due to different screening strategies, due to different TSH cut off values and genetic pattern of the mutated subjects. Only primary TSH-based screening programs are able to detect these patients, and only the use of low TSH spot threshold allows the detection of more cases with mild thyroid dysfunction generally associated with monoallelic defects (56,57). In infants with normal sized glands, the partial TSH resistance must be differentiated from mild forms of CH due to dyshormonogenetic defects. This diagnosis should be confirmed by radioiodine uptake and perchlorate discharge test performed at the moment of diagnostic re-evaluation of CH in all subjects in whom LOF TSHR mutations have been excluded (58). The generalized form of hormone resistance named PHP1a can be rarely responsible for mild forms of CH detected in the neonatal period; in these cases, the high levels of PTH and the associated typical phenotype can lead to the correct diagnosis (see above).

Today, due to the increasing sensitivity of laboratory assays for TSH, the occurrence of hormonal findings suggestive of partial TSH resistance in infancy or in childhood is very frequent. Only a minority of these subjects will be carriers of LOF TSHR mutations, so the criteria of selection of the patients who are candidates for genetic analysis become very important. The possibility of a mild form of hypothyroidism in heterozygous subjects for a TSHR gene mutation determined an increase of genetic studies in patients not selected by neonatal screening with the consequent increase of mutation detection previously identified in a single family. There are many Italian studies on this topic which reached different conclusions, most likely due to different selection criteria applied to the population examined. Alberti et al (12) found 4/10 mutated subjects among pediatric patients selected for hyperthyrotropinemia. Camilot et al (6) identified only 13 subjects with heterozygous mutation in a wide cohort of pediatric patients (116) where hyperthyrotropinemia was discovered by chance (prevalence of 11%), and these data were confirmed by Calebiro et al (5) who reported a frequency of 11.8% in a cohort of 152 patients aged <18 years with nonautoimmune hyperthyrotropinemia (NAHT). Recently, Rapa et al (7) found a prevalence similar to Camilot et al in a cross-sectional study that enrolled 88 patients from different pediatric centres. In our study, we enrolled 38 patients followed in the same centre and carefully selected on the basis of their history, hormonal and US parameters and prolonged clinical observation. We found a higher prevalence of 29% of subjects with heterozygous mutation (11/38) (8). 

In these subjects, besides the evaluation of the above-mentioned diseases, the diagnostic work-up must exclude AITD as the most frequent potential cause of subclinical hypothyroidism in late infancy and childhood. The differential diagnosis is based on the clinical history (in most cases, these patients show a progressive evolution from subclinical to overt hypothyroidism over time), the positivity of anti-thyroid antibodies and/or the finding of the typical non-homogeneous hypoechoic pattern at thyroid US.

Therefore, partial TSH resistance due to LOF TSHR mutations must be suspected in all subjects with:

1) TSH serum levels persistently above the upper limit of normal range or fluctuating around the upper limit of normal range with tendency to increase when increased thyroid activity is required (infancy, adolescence, pregnancy) 

2) normal/near-normal fT4 concentrations

3) absence of anti-thyroid antibodies

4) normal or hypoplastic thyroid gland in situ at US with normoechoic pattern 

5) positive family history for thyroid pathology (all reported TSHR gene mutations are familial but this does not exclude the possibility of de novo TSHR gene mutation)

4) absence of other defects/malformations typical of syndromic CH or PHP1a

5) negative perchlorate discharge test in selected cases

**When to Treat**

An early substitution treatment with Levo-T4 (L-T4) should be mandatory in all patients with severe CH due to complete uncompensated TSH resistance diagnosed at birth by neonatal screening. Recently, Dimitropoulos et al (59) reported mental impairment in children affected even when treated early with high doses of L-T4; therefore, in families at risk, the analysis of TSHR gene should be advisable for genetic counselling and possible prenatal diagnosis.

The usefulness of substitution treatment appears to be much more controversial in patients with subclinical hypothyroidism due to partial TSH resistance in whom the increased TSH concentration should be able to compensate the mild functional impairment of the mutant receptor. Certainly, there are many reports of both biallelic and monoallelic TSHR mutations diagnosed during childhood and adulthood by chance in patients who showed normal physical and neurological development (8,12,28,29). However, the great phenotypic variability observed also in siblings with the same mutation creates difficulties in clinical management and the need for treatment may depend not only on the severity of the impairment of the mutant receptor function but also on other factors such as concomitant thyroid disease or age. Particularly in pediatric patients, data from the literature are poor and not conclusive because of the possible negative effects caused by even mild hypothyroidism on cerebral development in this critic period of life (60,61).

Since TSH is thought to be the most sensitive and early indicator of insufficient thyroid production, there is general agreement among pediatric endocrinologists to consider a TSH serum value higher than 10 mU/L (after 2 weeks of life) as a criterium for beginning substitution therapy. In our study, following these criteria, L-T4 therapy was started in 6 of 16 subjects with TSHR gene mutations and in 8 of 27 subjects with similar NASH phenotype but without TSHR mutations (37.5 vs. 29.6%; NS). None of the treated children showed clinical signs of hypothyroidism, but in some parents, carriers of the mutation, the treatment became necessary for the onset of clinical features of hypothyroidism concomitant with AITD (8). Recently, two prospective longitudinal studies evaluated the clinical and hormonal course over time of patients with different LOF TSHR mutations with discordant results (24,53). Tenenbaum-Rakover et al (24) examined 33 subjects who were homozygotes, compound heterozygotes or heterozygotes for two TSHR gene mutations (P68S and L653V) and who presented with compensated hyperthyrotropinemia. With the exception of one individual with concomitant AITD, in all patients, examined TSH and T4 concentrations remained stable over time. 

Mizuno et al (53) reported the clinical and mental outcome of 5 Japanese patients with neonatal hyperthyrotropinemia in whom a homozygous R450H mutation of TSHR gene had been demonstrated. All patients were treated despite the persistent normal thyroid hormone levels and an increased dose of L-T4 was necessary to maintain the TSH levels within the normal range. Thyroid dysfunction with low T4 levels became obvious in one patient after interruption of medication in adolescence, although his thyroid hormone levels had been within the normal range in infancy. The IQ scores at 6 years of age were normal in all patients.

Obviously, more prospective longitudinal studies are needed to clarify the genotype-phenotype correlation in patients with partial TSH resistance and to improve the therapeutic approach. Today, the choice of treat or not to treat must be individualized according to biochemical and clinical parameters. In any case, a careful follow-up is needed in all untreated cases because some patients with fully or partially compensated TSH resistance could develop uncompensated resistance over time, in particular with concomitant AITD (2,53).

In summary, a review of available data on patients with loss-of-function mutations of TSHR genes shows that: 1) A mutation in the TSHR gene is a rare event in terms of mutated alleles/health alleles but may be a frequent cause of NAHT when the patients are accurately selected; 2) Together with standard criteria (TSH serum values above the upper limit of the normal range in two or more evaluations, normal fT4 serum values, absence of anti-thyroid antibodies, normal or hypoplastic thyroid gland in situ at US with a normoechoic pattern) also an accurate clinical work-up is needed to select patients who are candidates for a LOF TSHR mutation testing; 3) The need for substitution therapy with L-T4 remains controversial in particular in pediatric patients with partial TSH resistance. A careful long-term follow-up is recommended in these cases.

## Figures and Tables

**Table 1:**
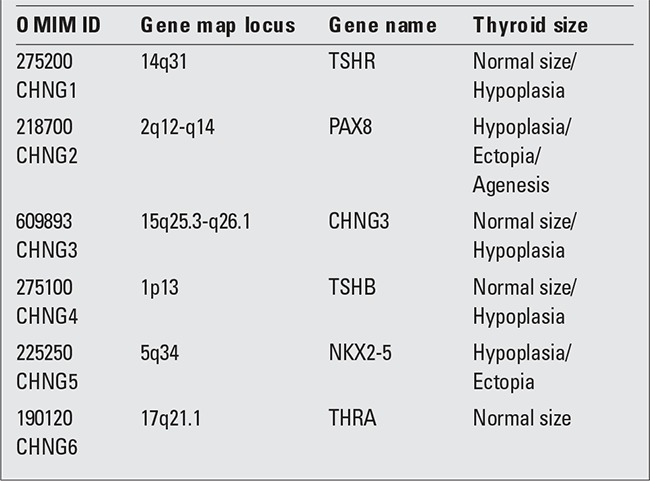
Classification of genetic forms of non-goitrous congenital hypothyroidism

**Table 2 continued 1:**
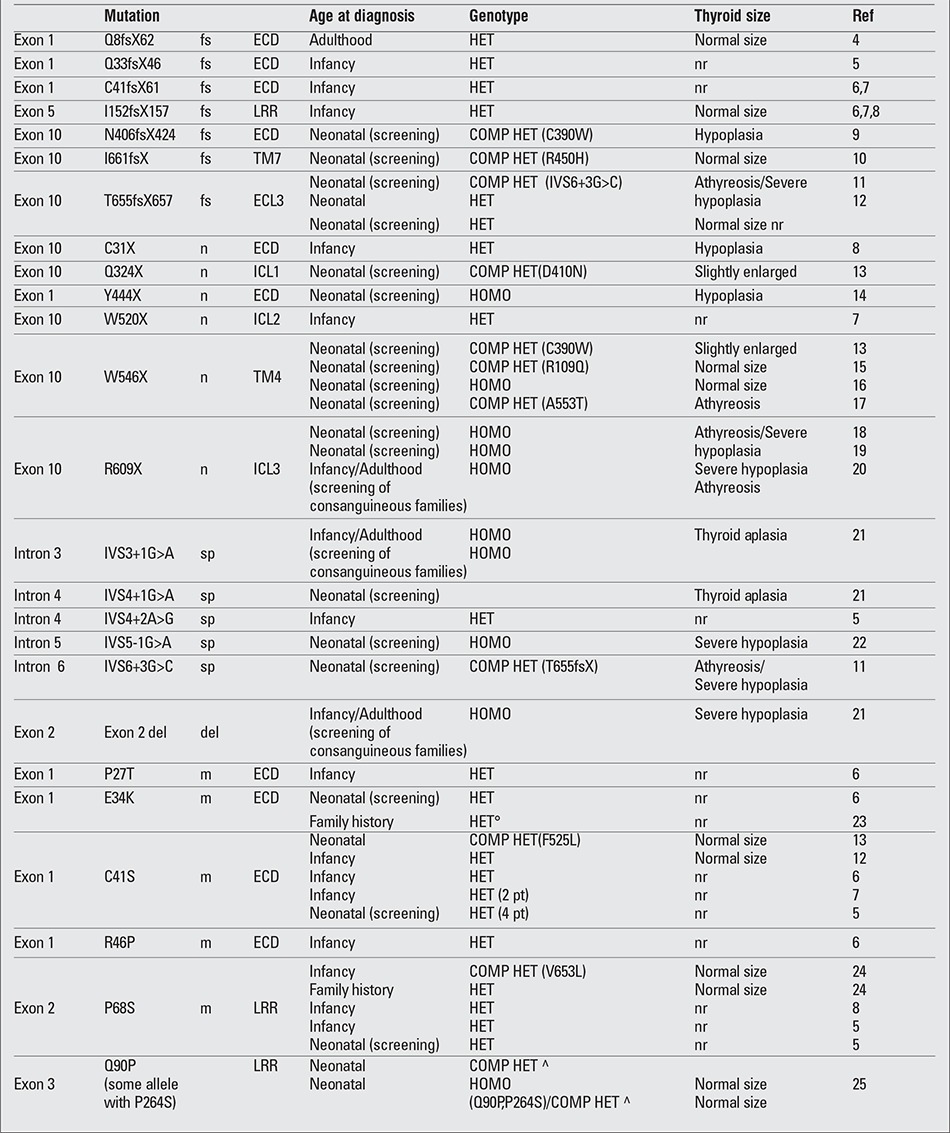
Spectrum of loss-of-function mutations in the thyroid stimulating hormone (TSH) receptor gene described to date

**Table 2 continued 2:**
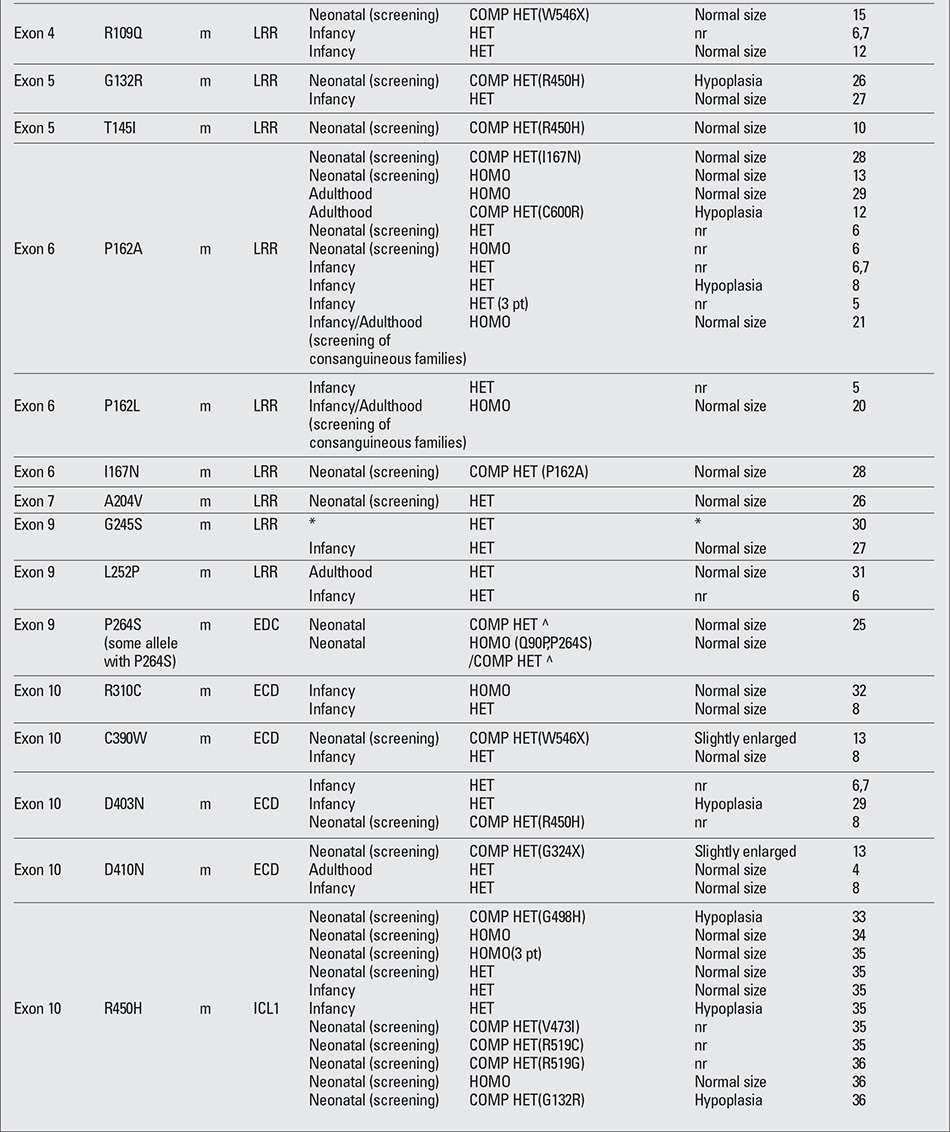
Spectrum of loss-of-function mutations in the thyroid stimulating hormone (TSH) receptor gene described to date

**Table 2 continued 3:**
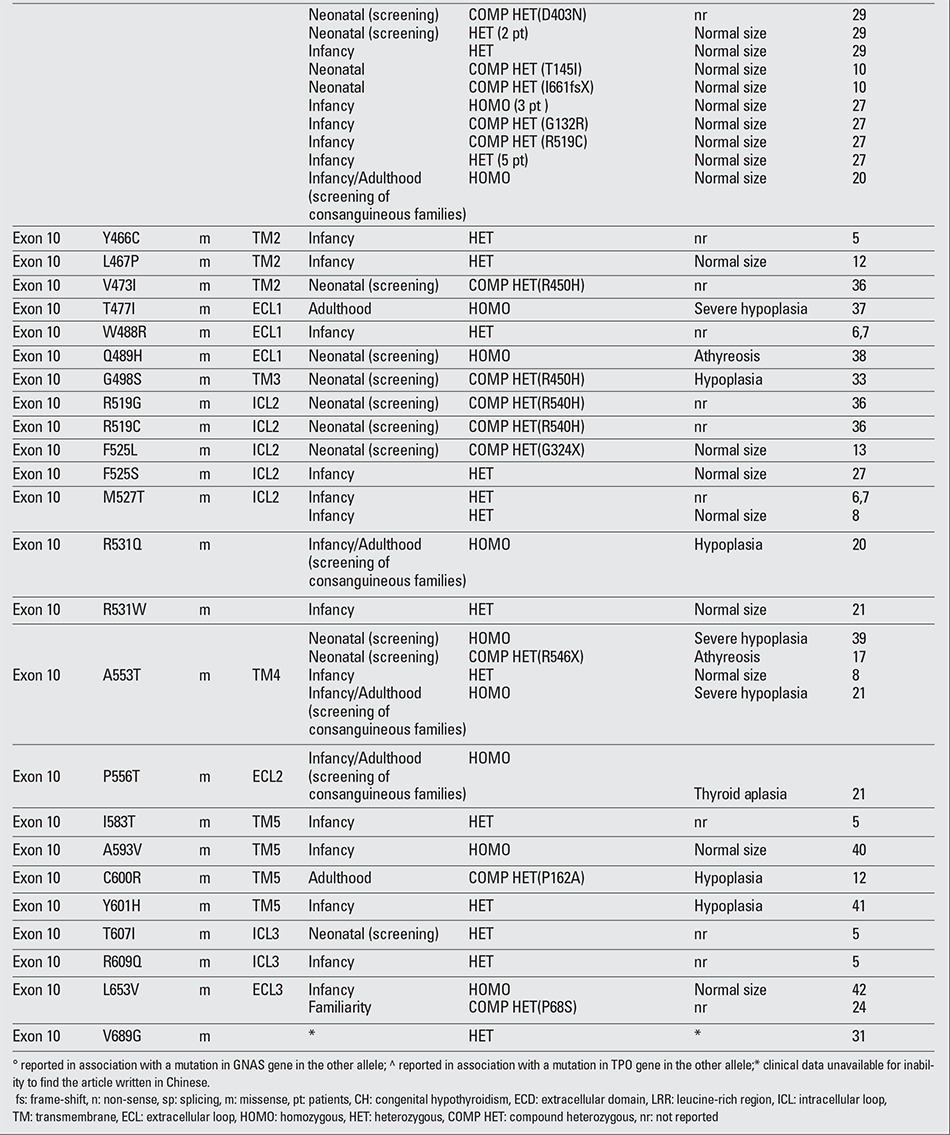
Spectrum of loss-of-function mutations in the thyroid stimulating hormone (TSH) receptor gene described to date

**Table 3:**
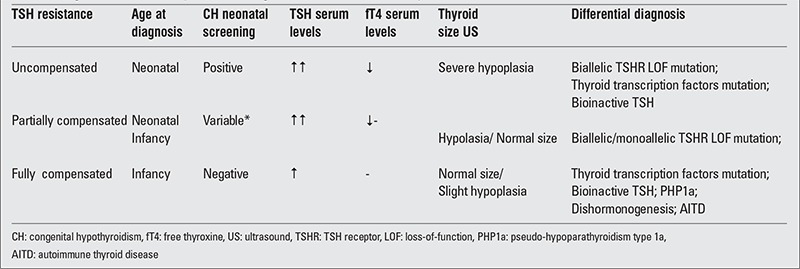
Diagnostic criteria for thyroid stimulating hormone (TSH) resistance syndromes
